# Dr. Cleverly

**Published:** 1825-01

**Authors:** 


### OBITUARY.

Dr. Cleverly.—Most of our readers are already acquainted with the premature decease of this gentleman; and we shall, therefore, content ourselves with laying before them a copy of the circular, which we have received in common with others of our professional brethren; and we shall feel happy if the additional publicity which the case thus acquires, has any effect in furthering the laudable objects of this appeal to the public.

"Among the numerous cases of distress, to which the attention of benevolent persons has been solicited, few have occurred more deplorable than that occasioned by the death of Dr. Cleverly, who was cut off in the prime of life, leaving a wife and five sons (the eldest fourteen,) utterly in a destitute state.

"This gentleman fell a victim to a fever of only five days' duration, which attacked him while engaged in his professional duties.

"His early prospects were blighted by his detention in France for eleven years; during which his kindness to his fellow prisoners, both professionally and in a pecuniary point of view, was unremitting.

"Dr. Cleverly, who was physician to the Orphan Asylum, as well as the Fever Hospital, was a licentiate of the College of Physicians, and highly esteemed by his professional brethren.

"Although this appeal is made to the feelings of medical man, the friends of the family trust that the public at large will be interested in the case of Dr. Cleverly, who died in their service."

---

